# Acetolactate Synthase-Inhibitor Resistance in *Monochoria vaginalis* (Burm. f.) C. Presl from Indonesia

**DOI:** 10.3390/plants11030400

**Published:** 2022-01-31

**Authors:** Ryan Widianto, Denny Kurniadie, Dedi Widayat, Uum Umiyati, Ceppy Nasahi, Santika Sari, Abdul Shukor Juraimi, Hisashi Kato-Noguchi

**Affiliations:** 1 Department of Agronomy, Faculty of Agriculture, Universitas Padjadjaran, Jl. Raya, Bandung Sumedang Km 21, Jatinangor, Sumedang 45363, Jawa Barat, Indonesia; ryanwidianto21@hotmail.com (R.W.); widayatdedi@yahoo.com (D.W.); umiyati_uum@yahoo.com (U.U.); santika@unpad.ac.id (S.S.); 2 Department of Pest and Diseases, Faculty of Agriculture, Universitas Padjadjaran, Jl. Raya, Bandung Sumedang Km 21, Jatinangor, Sumedang 45363, Jawa Barat, Indonesia; ceppynasahi@yahoo.com; 3 Department of Crop Science, Faculty of Agriculture, Universiti Putra Malaysia, Serdang 43400, Selangor, Malaysia; ashukor@upm.edu.my; 4 Department of Applied Biological Science, Faculty of Agriculture, Kagawa University, Miki 761-0795, Kagawa, Japan

**Keywords:** acetolactate synthase, herbicide resistance, *Monochoria vaginalis*, DNA, resistant spectrum

## Abstract

*Monochoria vaginalis* (Burm. f.) C. Presl, belonging to the family Pontederiaceae, is an aquatic herbaceous plant, native to temperate and tropical Asia. The species often occurs in paddy fields as a noxious weed in East Asia, and in the USA, and causes a significant reduction in rice production. The objective of the present research was the evaluation of the resistance levels of *M. vaginalis* against three chemical groups of acetolactate synthase (ALS)-inhibitor herbicides and other two different groups of herbicides, and the investigation of the mutations in the *ALS* gene of the resistant biotype of *M. vaginalis*. Herbicide dose–response experiments showed that the resistant biotype of *M. vaginalis* was highly resistant to bensulfuron-methyl, moderately resistant to bispyribac-sodium, had low resistance to penoxsulam and 2,4-D dimethyl ammonium, and was susceptible to sulfentrazone. The nucleotide sequences of the *ALS gene* of resistant and susceptible biotypes showed 14 base substitutions, which caused two amino acid substitutions: Val-143-Ile and Val-148-Ile. It is the first report of the substitutions of amino acids Val-143-Ile and Val-148-Ile in ALS protein. Those mutations may give different resistance spectra against three ALS-inhibitor herbicides: bensulfuron-methyl, bispyribac-sodium, and penoxsulam. Further research is needed to elucidate the molecular basis of target-site resistance mechanisms such as the transformation of the *ALS* gene of *M. vaginalis*. It is also necessary to evaluate herbicide mixtures and/or the rotation of herbicide sites of action to control the resistant biotype of *M. vaginalis.*

## 1. Introduction

Acetolactate synthase (ALS, EC 2.2.1.6, synonym: acetohydroxyacid synthase) catalyses pyruvate to acetolactate, which is the first step in the biosynthesis of the branched-chain amino acids (valine, leucine and isoleucine) [1]. The blocking of the biosynthesis causes subsequent plant death, and ALS is the target site for a large number of ALS-inhibitor herbicides [1,2]. ALS-inhibitor herbicides have been used for over 40 years, and more than 50 active ingredients are registered [1,2,3,4]. Those herbicides are divided into five chemical groups: sulfonylurea (SU), imidazolinone (IMI), pyrimidinyl thiobenzoate (PTB), sulfonylurea (SU), sulfonylamino-carbonyltriazonone (SCT), triazolopyrimidine (TP) [1,2].

The ALS-inhibitor herbicides have an efficient broad-spectrum in grass and broadleaf weed species, a low mammalian toxicity, and are selective in major crops in the world [1,3,4]. Therefore, the ALS-inhibitor herbicides are applied intensively over large areas in many different crop cultivations for weed management. However, excessive application of herbicides increases the potential to develop resistant biotypes of weeds [2,5,6]. The first case of an ALS-inhibitor resistance was reported in the population of *Lolium rigidum* Gaud. in Australia in 1986. This resistant biotype was caused by the enhanced metabolism of SU herbicide [7]. The first case of a target site resistance to an ALS-inhibitor herbicide was reported on *Lactuca serriola* L. against SU herbicide in the USA in 1987 [8]. There have been reported more than 160 ALS-inhibitor-resistant weed species, and some of those weed species are cross- and multiple-resistant to the herbicides, indicating that they are more difficult to control by the use of herbicides [1,9].

*Monochoria vaginalis* (Burm. f.) C. Presl (Pontederiaceae) is an aquatic herbaceous plant, native to temperate and tropical Asia, and predominantly self-pollinating with frequent cleistogamous reproduction [10]. The species often occurs in paddy fields as a noxious weed in East Asia [11], and in the USA [12,13]. It is listed in one of the three serious weeds in rice paddies in Indonesia, Japan and Taiwan [12,13]. As the species has a strong ability to absorb nitrogen, it causes a large reduction in rice production [11,14].

ALS-inhibitor herbicides are widely used in rice cultivation as pre-emergence herbicides [3]. Those herbicides were also effective in controlling *M. vaginalis.* However, the excessive application of the herbicides increases the potential to develop resistant weeds. SU-resistant biotypes of *M. vaginalis* have been reported in Japan, Korea and China [9], and an SU-resistant biotype of *M. vaginalis* has recently been found in Indonesia [15]. In the present research, we examined the tolerance of the SU-resistant biotype *Monochoria vaginalis* against different chemical groups of ALS-inhibitor herbicides (bensulfuron-methyl, bispyribac-sodium, and penoxsulam) and other herbicides which have another mode of action (2,4-D and sulfentrazone). The nucleotide sequences of the *ALS gene* of the resistant and susceptible biotypes of *M. vaginalis* were also determined.

## 2. Results

**Figure 1:**
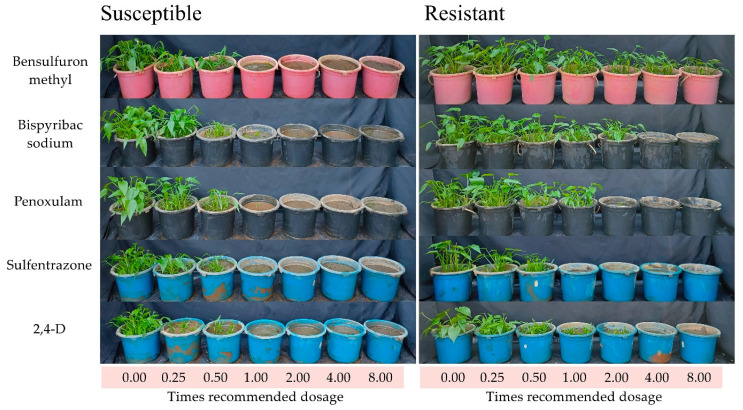
Effects of herbicides on susceptible and resistant biotypes of *M. vaginalis.* Herbicides were applied at seven dosage levels (0, 0.25, 0.5, 1, 2, 4, and 8 times of herbicide recommended dosage) at 2 weeks after sowing of *M. vaginalis*: bensulfuron-methyl (0, 1, 2, 4, 8, 16, 32 g a.i. ha^−1^), penoxsulam (0, 2.5, 5, 10, 20, 40, 80 g a.i. ha^−1^), bispyribac-sodium (0, 5, 10, 20, 40, 80, 160 g a.i. ha^−1^), sulfentrazone (0, 12, 24, 48, 96, 192, 384 g a.i. ha^−1^), and 2,4-D dimethyl ammonium (0, 108.12, 216.25, 432.5, 865, 1730, 3460 g a.i. ha^−1^). Photos were taken 28 days after herbicide application.

For the overall analysis of variance, all herbicides showed significant interactions at *p* < 0.05 level for growth reduction and shoot dry weight of *M. vaginalis.* The application of recommended dosages of bensulfuron-methyl (4 g a.i. ha^−1^), penoxsulam (10 g a.i. ha^−1^), bispyribac-sodium (20 g a.i. ha^−1^), sulfentrazone (48 g a.i. ha^−1^), and 2,4-D dimethyl ammonium (432.5 g a.i. ha^−1^) cause 100% growth reduction of the susceptible biotype of *M. vaginalis* (Figure 1, Table 1). The recommended dosages of sulfentrazone also cause 100% growth reduction of the resistant biotype of *M. vaginalis*. However, the recommended dosages of bensulfuron-methyl, penoxsulam, bispyribac-sodium, and 2,4-D could not cause 100% growth reduction on the resistant biotype. The growth reduction by the recommended dosages was 18.98, 34.10, 22.72, and 69.50% for bensulfuron-methyl, penoxsulam, bispyribac-sodium, and 2,4-D, respectively. The complete growth reduction of the resistant biotype occurred when penoxsulam, bispyribac-sodium, and 2,4-D were applied in doses 2–4 times higher than the recommendation. The dosage (32 g a.i. ha^−1^) of bensulfuron-methyl, which is eight times higher than the recommendation, caused the 37.27% growth reduction (Table 1).

**Figure 2:**
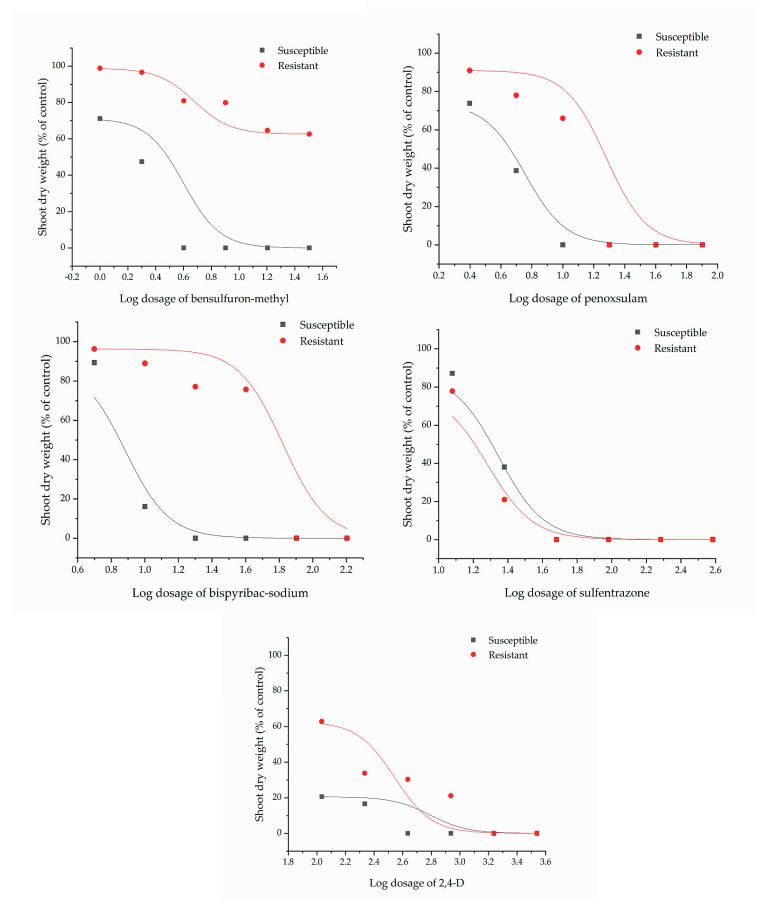
Growth reduction curves of susceptible and resistant biotypes of *M. vaginalis* by herbicide application. Dosage indicated times of recommended dosage as described in Figure 1.

The GR_50_ values of bensulfuron-methyl, penoxsulam, bispyribac-sodium, sulfentrazone, and 2,4-D for the resistant and susceptible biotypes of *M. vaginalis* were determined as Figure 2, and shown in Table 2. The ALS-inhibitor herbicides bensulfuron-methyl, penoxsulam, and bispyribac-sodium caused the symptoms of necrosis, chlorosis, stunting, and purple veins in *M. vaginalis* plants. 2,4-D showed the symptoms of twisting, leaf curling, and cupping. Sulfentrazone induced the symptoms of chlorosis and necrosis in the plants.

**Figure 3:**
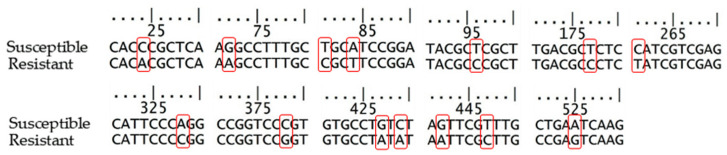
Alignment of partial nucleotide sequences of *ALS gene*. Red boxes indicate the substitution.

The base sequence of PCR-amplified DNA showed complete homology with that of *ALS* genes of *M. vaginalis* [16]. The base sequences showed 14 differences in nucleotides between susceptible and resistant biotypes at the positions of 24,72, 81, 84, 96, 177, 261, 328, 378, 427, 429, 442, 447, and 525 (Figure 3).

**Table 2:** Herbicide dose required for 50% reduction (GR_50_) of dry biomass, and resistance index.

Herbicide	*b*	*r^2^*	*M. vaginalis* Biotypes	GR_50_ (g a.i ha^−1^)	R/S	Resistance Category [17]
Bensulfuron-methyl	2.45	0.94	Susceptible	1.66	-	-
	0.72	0.87	Resistant	51.93	31.28	High
Penoxsulam	2.69	0.97	Susceptible	3.93	-	-
	3.78	0.93	Resistant	11.03	2.81	Low
Bispyribac-sodium	5.50	0.99	Susceptible	7.41	-	-
	6.82	0.91	Resistant	47.03	6.35	Moderate
Sulfentrazon	3.79	0.99	Susceptible	20.83	-	-
	3.79	0.99	Resistant	16.78	0.81	Susceptible
2,4-D	1.28	0.82	Susceptible	40.79	-	-
	1.07	0.91	Resistant	158.27	3.88	Low

GR_50_ values of herbicides were obtained by nonlinear regression using the log-logistic dose–response equation. The resistance index against herbicides was calculated by the ratio of the GR_50_ value of the resistant biotype to susceptible biotype of *M. vaginalis*.

## 3. Discussion

The resistant biotype of *M. vaginalis* showed tolerance against bensulfuron-methyl, bispyribac-sodium, penoxsulam, and 2,4-D dimethyl ammonium (Figure 1, Table 1). The level of resistance index (R/S) was calculated by the ratio of their GR_50_ values of resistant biotype (R) with susceptible biotype (S), and classified as susceptible (R/S < 2), low resistance (R/S = 2–6), moderate resistance (R/S = 6–12), and high resistance (R/S > 12) [17]. The resistant biotype of *M. vaginalis* showed different resistance spectra in response to different groups of ALS-inhibitor herbicides: it was highly resistant to bensulfuron-methyl (R/S: 31.28, SU herbicide group); moderately resistant to bispyribac-sodium (R/S: 6.35, PTB herbicide group); had low resistance to penoxsulam (R/S: 2.81, TP herbicide group). The biotype also showed low resistance to 2,4-D (R/S: 3.88, synthetic auxin group), which has a different mode of action with ALS-inhibitor herbicides. 2,4-D disturbs several growth processes and affects nucleic acid metabolism, cell wall plasticity, protein synthesis, and cell division. The herbicide also enhances ethylene production, which often causes epinastic symptoms [18]. The resistant biotype was susceptible to sulfentrazone (R/S: 0.81, triazolinone group). Sulfentrazone is also used as a broad-spectrum herbicide. It is an inhibitor of the protoporphyrinogen oxidase (PPO, EC 1.3.4.1.) involved in protoporphyrin biosynthesis, which is a precursor of chlorophylls. The inhibition of PPO also results in forming highly reactive molecules that destroy cell membranes. Visible symptoms of sulfentrazone are chlorosis and desiccation [19,20].

The resistant biotype of *M. vaginalis* showed cross-resistance against three different chemical groups of ALS-inhibitor herbicides, and multiple resistance against two different herbicide groups which have different modes of action. SU-resistant biotypes of *M. vaginalis* were first observed in rice fields in Japan in 1989 [21]. Further SU-resistant biotypes of *M. vaginalis* were found in rice fields in Korea in 1990 and China in 2010 [9]. Resistant biotypes found in Korea also showed cross-resistance against different chemical groups of ALS-inhibitor herbicides, pyrazosulfuron-ethyl (SU group), bensulfuron-methyl (SU group), cyclosulfamuron (SU group), and flumetsulam (TP group) [22]. However, the 2,4-D-resistant biotype of *M. vaginalis* was observed in this research for the first time.

**Figure 4:**
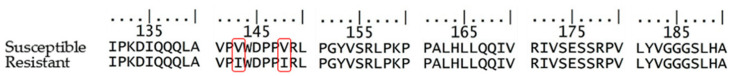
Alignment of deduced amino acid sequences of *ALS gene*. Red boxes indicate the substitution.

Among 14 base differences in *ALS genes* of resistant and susceptible biotypes of *M. vaginalis* (Figure 3), 427-429 (GTC; susceptible, ATA; resistant) and 442-445 (GTT; susceptible, ATT; resistant) cause two amino acid substitutions: Val-143-Ile and Val-148-Ile (Figure 4). The Pro-197-Ser amino acid substitution was found in resistant biotypes of *M. vaginalis* against ALS-inhibitor SU herbicide [21,23,24]. There have been 27 types of amino acid substitutions reported in resistant weeds against ALS-inhibitor herbicides. Among them, 11 amino acid substitutions generated highly resistant biotypes and another 16 amino acid substitutions caused moderately resistant biotypes [1,9]. The substitution of Pro-197-Ser was caused by only one nucleotide mutation, and the most abundant. Those mutations gave different resistance spectra against SU and TP herbicides [1,9]. Substitutions of two amino acids in ALS protein were reported in this research for the first time: Val-143-Ile and Val-148-Ile.

ALS has a regulatory subunit and a catalytic subunit. The regulatory subunit controls the activity of the catalytic subunit, including the feedback inhibition by produced amino acids such as valine, leucine, and isoleucine. It has been found that the herbicides cannot mimic the substrate for the enzyme based on the three-dimensional structure analysis of ALS, substrate and herbicide molecules. Thus, ALS-inhibitor herbicides do not bind with the catalytic site of the catalytic subunit, but bind across the catalytic site and interfere with the access of the substrate to the catalytic entry point of ALS [25]. The resistant biotype of *M. vaginalis* showed differing resistance levels to different chemical groups of ALS-inhibitor herbicides (Table 2). Those herbicides may bind differently across the catalytic site of ALS protein, which may cause the different efficiency for those herbicides to block substrate to access to the catalytic entry. It was also reported that two chemical groups of ALS-inhibitor herbicides, SU and TP herbicides bound different sites of ALS protein with partially overlapping [25]. However, it has been reported that four *ALS* genes exist in *M. vaginalis* [21]. Thus, the mutations of Val-143-Ile and Val-148-Ile should be examined for each *ALS* gene.

The resistant biotype of *M. vaginalis* showed low resistance to 2,4-D. Synthetic auxin herbicides were introduced in the 1940s, and more than 30 resistant weed species were reported. However, only a few resistant species are widespread, and the synthetic auxin herbicides remain effective to control several weeds [2]. The resistance mechanism against 2,4-D has been reported in only a few species. The resistant biotype of *Galeopsis tetrehit* L., which is resistant to the herbicide 4-chloro-2-ethyphenoxyacetate (MCPA), has the gene mutations involved in the reduction of 4-chloro-2-ethyphenoxyacetate (MCPA) translocation, and activation of MCPA degradation metabolism [26]. It is necessary to elucidate the biochemical and molecular basis of evolved resistance of *M. vaginalis* to synthetic auxin herbicides in the future.

The appearance of multiple resistance and cross-resistance in weeds is a serious issue for weed control. The resistant biotype of *M. vaginalis* showed tolerance against three chemical groups of ALS-inhibitor herbicides (bensulfuron-methyl, bispyribac-sodium and penoxsulam), and a synthetic auxin herbicide (2.4-D). However, the resistant biotype was susceptible to a PPO-inhibitor herbicide (sulfentrazone). The most effective herbicide-resistance management strategy is probably to rotate herbicide sites of action [27,28]. The employment of herbicide mixtures, such as combinations of PPO-inhibitor and other herbicides, and the rotation of different types of herbicides in different growing seasons, may be an option to control *M. vaginalis* resistant biotypes [2,9,29].

## 4. Materials and Methods

### 4.1. Plant Materials

Susceptible and resistant biotypes of *M. vaginalis* seeds were collected from lowland rice cultivation areas in March 2021. The susceptible biotype was collected from Kertajaya Village, Tambakdahan District, Subang Regency, West Java Province (6°21′39.9″ S 107°48′07.8″ E). The resistant biotype was collected from Kalentambo Village, Pusakanegara District, Subang Regency, West Java Province (6°15′32.8″ S 107°53′34.6″ E). The resistant population of *M. vaginalis* has been identified as bensulfuron-methyl resistant biotype [15]. The collected seeds were then cleaned and dried in the sun for one week to reduce their moisture and increase the maturity.

### 4.2. Herbicide Dose–Response Experiments

Herbicide dose–response experiments were carried out using the whole plant pot test dose–response method [30]. Pots (20 cm in diameter) were filled with paddy soil (3 kg) after sterilisation using an autoclave at 120 °C and 15 psi for 2 h so that only *M. vaginalis* seeds could be allowed to germinate and grow. Then, 10–20 seeds of *M. vaginalis* were sowed on the soil surface in the pots. Sufficient water was supplied to the pots, and the water level was maintained at 0.5 cm above the soil surface during the experiments. Two weeks after sowing (1–2 leaf stage of *M. vaginalis*), herbicides were applied at seven dosage levels (0, 0.25, 0.5, 1, 2, 4, and 8 times of herbicide recommended dosage): bensulfuron-methyl (0, 1, 2, 4, 8, 16, 32 g a.i. ha^−1^; Benson 10 wp, PT Dalzon, Wangunharja, Indonesia), penoxsulam (0, 2.5, 5, 10, 20, 40, 80 g a.i. ha^−1^; Clipper 25 OD, Corteva, Wilmington, USA), bispyribac-sodium (0, 5, 10, 20, 40, 80, 160 g a.i. ha^−1^; Tabbas 400 SC, Deltagro Mulia Sejati, Jakarta, Indonesia), sulfentrazone (0, 12, 24, 48, 96, 192, 384 g a.i. ha^−1^; Boral 480 SC, PT. Bina Guna Kimia, Jawa Tengah, Indonesia), and 2,4-D dimethyl ammonium (0, 108.12, 216.25, 432.5, 865, 1730, 3460 g a.i. ha^−1^; Indamin 865 SL, PT. Indagro, Jakarta, Indonesia). Herbicide application was carried out with a spray volume of 400 L ha^−1^ using a semi-automatic knapsack sprayer with a flat fan nozzle at a pressure of 138 kPa. *M. vaginalis* was harvested 28 days after herbicide application, and five plants for each treatment were used for the determination of their mass.

### 4.3. Statistical Analyses for the Dose–Response Experiments

The experimental design used in this experiment was a 2-factor split-plot with three replications. The percentage of growth reduction data was obtained from the comparison between the dry weight of herbicide application weeds (T) and the dry weight of control weeds, to which no herbicide was applied (C), using the following equation [31]: The growth reduction (%) = [1 − (T/C)] × 100. The growth reduction was transformed using the square root transformation (square root of data + 0.5) and then analysed using ANOVA. The interaction between weed populations and herbicide doses was analysed based on a *p*-value < 0.05. If an interaction occurred, Tukey’s test was used to identify significant differences.

The herbicide doses required for 50% growth reduction (GR_50_) were obtained by nonlinear regression using the log-logistic dose–response equation [32]: Y = c + (d − c)/[1 + (X/GR_50_)^b^], where c and d denote lower and upper limits, respectively, and b is the slope of the response curve. The percentage of damage, obtained from the conversion of weed dry weight yield, was used to determine the GR_50_. Ideally, the doses used should cover the whole range of responses, from no visible effects to complete weed mortality. Dose–response analysis was performed using *OriginPro* 9.0 software.

### 4.4. Isolation of DNA and Gene Sequence

Genomic DNA was isolated from the leaves of susceptible and resistant biotypes of *M. vaginalis* using “Plant Genomic DNA Mini Kit” (GP-100, Geneaid Biotec Ltd., Taipei, Taiwan). A primer set 5′-ATGGCTGCTTCGAAGCCCTCTCCATT-3′ (forward) and 5′-ACTAGTGCACTGTGCTCCCATCTCCAT-3′ (reverse) [24] was used for PCR amplification of *ALS* genes of *M. vaginalis.* The amplification was performed in a total volume of 50 µL containing 25 µL “KOD One PCR master mix” (KMM-201 Toyobo Co., Ltd., Osaka, Japan), 1.5 µL each primer (0,3 µM), 21 µL dd H_2_O, and 1 µL DNA of *M. vaginalis* (10 ng). The PCR conditions were as follows: denaturation at 94 °C for 2 min; 35 cycles of 98 °C for 15 s, 64 °C for 30 s; and final extension at 68 °C for 45 s. After the amplification process, the DNA sequence process was carried out using the capillary electrophoresis method (Sanger sequencing method) [33] with a thermal cycler: Agilent Surecycler 8800 (Agilent Technologies Inc., Santa Clara, CA, USA). The experiment was repeated three times independently to confirm the validity.

## 5. Conclusions

The resistant biotype of *M. vaginalis* showed cross-resistance at different levels against three different chemical groups of ALS-inhibitor herbicides: bensulfuron-methyl (SU), bispyribac-sodium (PTB), and penoxsulam (TP). The biotype also showed resistance to 2,4-D, which has a different site of action from that of ALS-inhibitor herbicides. However, it is susceptible to sulfentrazone. Amino acid substitutions of ALS protein of the resistant biotype were found in Val-143-Ile and Val-148-Ile. Those mutations may give different resistance spectra against three ALS-inhibitor herbicides. However, it is necessary to elucidate the molecular basis of target-site resistance mechanisms such as the transformation of the *ALS* gene of *M. vaginalis*. The employment of herbicide mixtures and/or the rotation of herbicide sites of action should be considered in order to control the biotype of *M. vaginalis.*

## Figures and Tables

**Table 1:** Effects of herbicides on the growth reduction (%) of susceptible and resistant biotypes of *M. vaginalis*.

Herbicide	Biotype	Herbicides Dosages (Times of Recommended Dosage)						
		0	0.25	0.5	1	2	4	8
Bensulfuron-methyl	Susceptible	0.00 a,D	28.42 a,C	52.54 a,B	100 a,A	100 a,A	100 a,A	100 a,A
	Resistant	0.00 a,C	1.10 b,C	3.49 b,C	18.98 b,B	19.84 b,B	35.22 b,A	37.27 b,A
Penoxsulam	Susceptible	0.00 a,D	25.85 a,C	60.87 a,B	100 a,A	100 a,A	100 a,A	100 a,A
	Resistant	0.00 a,E	8.87 b,D	21.72 b,C	34.10 b,B	100 a,A	100 a,A	100 a,A
Bispyribac-sodium	Susceptible	0.00 a,C	10.29 a,B	83.84 a,A	100 a,A	100 a,A	100 a,A	100 a,A
	Resistant	0.00 a,D	3.66 b,D	10.99 b,C	22.72 b,B	24.17 b,B	100 a,A	100 a,A
Sulfentrazone	Susceptible	0.00 a,D	12.61 b,C	61.76 b,B	100 a,A	100 a,A	100 a,A	100 a,A
	Resistant	0.00 a,D	22.11 a,C	79.10 a,B	100 a,A	100 a,A	100 a,A	100 a,A
2,4-D	Susceptible	0.00 a,C	79.41 a,B	83.35 a,B	100 a,A	100 a,A	100 a,A	100 a,A
	Resistant	0.00 a,E	37.02 b,D	66.08 b,C	69.50 b,C	78.76 b,B	100 a,A	100 a,A

Herbicides were applied at seven dosage levels (0, 0.25, 0.5, 1, 2, 4, and 8 times of herbicide recommended dosage) at 2 weeks after sowing of *M. vaginalis*, and *M. vaginalis* was harvested 28 days after herbicide application. The percentage was calculated from the comparison between the dry weight of herbicide-applied *M. vaginalis* and that of control *M. vaginalis*, with a value of 100% indicating complied inhibition. The values in each column followed by the same lowercase letters (vertical direction) and uppercase letters (horizontal direction) are not significantly different at *p* < 0.05 according to the Tukey Test for each herbicide.

## Data Availability

Not applicable.
